# The Poor and the Poor Law


**Published:** 1894-03-24

**Authors:** 


### Studies in Applied Sociology.

### V.—THE POOR AND THE POOR LAW.*

Thoughtless agitators, claiming as they say "not charity, but justice," are apt to speak of the poor law as an engine specially devised for the insult and degradation of the working classes; yet in the modern demand for State-provided labour at remunerative wages for those who can work, and a comfortable State-aided maintenance for those who cannot, they are only asking again what the poor law granted a hundred years ago, with what results to the working classes we desire very briefly to show.

It was not till the reign of Elizabeth that there was any poor law in England. Before the suppression of the monasteries the Church looked after the poor, and, it is to be feared, encouraged by indiscriminate charity the poverty it relieved. Failing the monasteries, the duty of providing for the poor fell on the parish, which raised funds to provide for the aged and infirm, apprenticed the children of needy parents, and provided work for the unemployed. It was, however, never intended to provide for those who could maintain themselves, or who had relatives to support them. The parish of birth was obviously the place in which relief could at the last resort be claimed, though subsequent legislation gave a "settlement," with claims upon the rates, of parishes where anyone had been apprenticed, had worked for a year, or in various other ways established a claim. This law of settlement became the fruitful parent of injustice and abuses. No man wishes to pay another man's debts; no parish wishes to support another district's poor. Therefore a law, due to the retrograde policy of the reign of Charles II., ordained that any stranger coming to a parish might be removed within forty days, unless he could give security that he would not become chargeable to the rates! The reason of this monstrous piece of legislation was avowedly that people moved to other parishes in order to better their condition; and this the statesmen of that day strove to discourage.

During this period it is undeniable that wrong was wilfully done to the poor; but a hundred years ago came the reaction. It is the result of this reaction we would commend to the notice of philanthropists. In 1795 the Berkshire magistrates resolved to make allowances for the relief of all poor and industrious men and their families. The example was followed elsewhere, and it is not too much to say that in the result the whole labouring class became pauperised. The allowance being proportioned to the size of a family, destroyed those prudential considerations which cause a couple to delay marriage until a competence is secured. Young people went straight from church to the overseer of the poor to demand a house from the public funds. Their children were practically supported by the rates from the moment of their birth. As for illegitimate children, as their mother received an allowance for each, the woman of loose character, when thus endowed, came to be regarded as a more desirable wife than her more virtuous but poorer sister; the poor law in this case actually led to the degradation of English womanhood.

In every instance the action of the new humanitarian spirit injured those whom it sought to serve. The result of the allowance to the industrious poor was to lower the rate of wages. With parish bread and parish money secured, they could afford to sell their labour at a smaller price. It is true that the labourer tried to cheat the overseer of the parish by stating the wages of the poorest week or month of the year as his average earnings, and had his allowance fixed accordingly; that he became improvident, and when he made large wages in the summer he spent them, and came on the rates in the winter. But with all the tricks of the willing pauper, he was no better off than he was before, while his action served to bring down to the level of starvation the more self-respecting workman who wished to keep himself. At this period the notion—not so new as its upholders think—of providing work for the unemployed was put into practice. Who benefited by it? The employers. Pauper labour was sold cheap. "In one place," says Mr. Fowle, "there was a weekly sale, where an eye-witness saw ten men knocked down for five shillings." Even if the farmer had to pay heavy poor rates to eke out such a wage, he bought his labour cheaper than if he had paid for it the proper price in open market. So well was this understood that if it was known that a workman had savings he was discharged till these were spent, and he could be sold into slavery as a pauper labourer. In short, so numerous and grave were the abuses of this law, that no less an economist than Malthus gave it as his opinion that if there had been no poor law, though there might have been keener cases of distress occasionally, the condition of the working classes as a whole would have been happier and more prosperous.

But the public conscience cannot endure the thought of these occasional cases, and so our poor laws are enacted. They are, practically, charitable in their action; but a careful distinction, which is pointed out by Mill, must be made between poor law relief and private benevolence. The latter may discriminate between case and case, and give more to the more deserving; such is not the function of the public almoner, who has nothing to do with merit, but only with need. Secondly, the private donor may give comforts, treats, luxuries, to the object of his beneficence; the public official, dealing with other people's money, and that money obtained by compulsion, is not justified in providing for more than the bare necessities of life. It is this distinction which forms the case against outdoor relief—the one form of relief acceptable to the poor. People who know that they will receive out-relief in their old age, do not struggle to save for that time and its needs. Children who will not have it said that their parents are in the workhouse, will accept a parochial subsidy for their maintenance; were they refused it, they would probably still manage to provide for the old people. Parochial relief is meant for the destitute, not for those whose

* See pamphlet "On the Development of the English Poor Law," by H. W. Hamilton Hoare (London: W. Ridgway, 169, Piccadilly, W.; price 6d.), and "The Poor Law," by the Rev. T. W. Fowle, M.A., Rector of Islip, in the "English Citizen" Series (Messrs. Macmillan and Co.; price 3s. 6d.).

kindred are able to support them. Out-relief should never be more than a grant-in-aid, as it sometimes is in the case of widows who can earn something, but not enough to maintain all the children dependent on them; though it has been found in many cases that even in such instances the usual result has followed—that the woman whom the parish helps will work for lower wages than another, and so injures her neighbours without bettering herself. But the workhouse test breaks up a family. In accepting indoor relief husband must part from wife, parent from child. This is used as an argument against it; but in truth it is not so. Outdoor relief breeds pauperism. Mr. Charles Booth's statistics of East London show how pauper children are born to pauper parents, who come upon the rates for the cost of their birth, and regard the parish as their destined providence. No one will pretend that this is desirable—that all the virtues which the responsibilities of family relations bring forth should be swamped by the lazy consciousness of even such a harsh foster-mother to bear our burdens. But few of us—nor proletarians more than peers—are willing to work for our living if someone else will do it for us, unless some penalty be attached to our idleness. The lot of the pauper must be less desirable than that of the poorest independent labourer, or we shall all be tempted to become paupers. And then where would the rates to support us be drawn from?

